# Olaparib-mediated enhancement of 5-fluorouracil cytotoxicity in mismatch repair deficient colorectal cancer cells

**DOI:** 10.1186/s12885-021-08188-7

**Published:** 2021-04-22

**Authors:** Helena de Castro e Gloria, Laura Jesuíno Nogueira, Patrícia Bencke Grudzinski, Paola Victória da Costa Ghignatti, Temenouga Nikolova Guecheva, Natalia Motta Leguisamo, Jenifer Saffi

**Affiliations:** 1 Laboratory of Genetic Toxicology, Federal University of Health Sciences of Porto Alegre (UFCSPA), Sarmento Leite st 245, Porto Alegre, RS Brazil; 2 Cardiology Institute of Rio Grande do Sul/ University Foundation of Cardiology (ICFUC), Porto Alegre, RS Brazil

**Keywords:** Colorectal cancer, Mismatch repair, 5-fluorouracil, PARP, Olaparib

## Abstract

**Background:**

The advances in colorectal cancer (CRC) treatment include the identification of deficiencies in Mismatch Repair (MMR) pathway to predict the benefit of adjuvant 5-fluorouracil (5-FU) and oxaliplatin for stage II CRC and immunotherapy. Defective MMR contributes to chemoresistance in CRC. A growing body of evidence supports the role of Poly-(ADP-ribose) polymerase (PARP) inhibitors, such as Olaparib, in the treatment of different subsets of cancer beyond the tumors with homologous recombination deficiencies. In this work we evaluated the effect of Olaparib on 5-FU cytotoxicity in MMR-deficient and proficient CRC cells and the mechanisms involved.

**Methods:**

Human colon cancer cell lines, proficient (HT29) and deficient (HCT116) in MMR, were treated with 5-FU and Olaparib. Cytotoxicity was assessed by MTT and clonogenic assays**,** apoptosis induction and cell cycle progression by flow cytometry, DNA damage by comet assay. Adhesion and transwell migration assays were also performed.

**Results:**

Our results showed enhancement of the 5-FU citotoxicity by Olaparib in MMR-deficient HCT116 colon cancer cells. Moreover, the combined treatment with Olaparib and 5-FU induced G2/M arrest, apoptosis and polyploidy in these cells. In MMR proficient HT29 cells, the Olaparib alone reduced clonogenic survival, induced DNA damage accumulation and decreased the adhesion and migration capacities.

**Conclusion:**

Our results suggest benefits of Olaparib inclusion in CRC treatment, as combination with 5-FU for MMR deficient CRC and as monotherapy for MMR proficient CRC. Thus, combined therapy with Olaparib could be a strategy to overcome 5-FU chemotherapeutic resistance in MMR-deficient CRC.

## Background

Colorectal cancer (CRC) is a major health problem worldwide owing to its high prevalence and mortality rates [1]. The standard regimen of CRC consists in 5-fluorouracil (5-FU)-based chemotherapy alone or in combination with oxaliplatin and/or irinotecan and targeted-therapies [2]. However, the improvements of these combinations in clinical outcomes are limited, mostly because of drug resistance [3]. Thus, the identification of biological factors to enhance the therapeutic response could improve survival rates in CRC patients.

The substantial progresses in the comprehension of CRC molecular complexity allowed the recent developments in the clinical management of this disease, including the identification of microsatellite instability (MSI) [4, 5]. MSI in sporadic CRC occurs as a result of random *hMLH1* promoter hypermethylation, which leads to the inactivation of mismatch repair (MMR) pathway [6]. MMR deficiency (dMMR) in early stage CRC predicts lower recurrence rates and partially justifies the absence of indication of fluoropyrimidine adjuvant monotherapy [7, 8]. In counterpart, patients harboring stage III dMMR/MSI tumors benefit from oxaliplatin-based adjuvant chemotherapy [9, 10].

The 5-FU-mediated cell death mechanisms include thymidylate synthase (TS) inhibition, dNTP pool imbalances and misincorporation of uracil and fluorouracil bases into DNA during replication. Proficient MMR (pMMR) CRC cells have increased sensitivity to 5-FU in comparison to dMMR CRC cells. The differences between these two molecular phenotypes occur due to a generation of a futile cycle of repair in the attempt to remove uracil and fluorouracil bases during DNA replication in pMMR CRC cells [11]. Base Excision Repair (BER) pathway also imposes its own toxicity, triggered by uracil–DNA glycosylase (UDG) and AP endonucleases [12–14]. Finally, if 5-FU is not removed from DNA, UDG creates lesions, such as abasic sites, single-strand DNA breaks (SSBs) and double-stranded DNA breaks (DSBs) that activate Homologous Recombination Repair (HRR) [15].

In this scenario, emerges the fundamental role of Poly (ADP-ribose) Polymerase-1 (PARP-1) that senses and binds SSBs, favoring the assemble of DNA repair effectors at the site of damage [16]. Besides its essential role in BER pathway, PARP-1 regulates replication fork progression [17]. Chemical inhibition of PARP1 impairs its catalytic activity, generating and impeding SSBs repair [18]. Persistence of SSBs stall and collapse replication fork resulting in DSBs [19]. HRR-deficient tumors are highly sensitive to PARP inhibitors (PARPis), such as Olaparib (AZD2281) [20–22].

PARP inhibition antineoplastic effects are not limited to HRR-defective tumors, and it is considered a radio- and chemosensitizer [23, 24].

It was recently proposed that replication stress-associated DSBs also induce MSI in dMMR cells and MSI tumors exhibit mutations within DNA repair genes [25], which could increase the susceptibility to PARP inhibition [26–29]. Although clinical relevance of MMR status in CRC prognosis and response to 5-FU is undeniable [30], information regarding efficacy of this agent and PARPis in CRC cells with different genetic backgrounds is still controversial [31–33]. Thus, the aim of this study was to evaluate the effect of the PARPi Olaparib on 5-FU cytotoxicity in MMR-deficient and proficient CRC cells and the mechanisms underpinning this response.

## Methods

### Reagents and drugs

Culture media and supplements (Dulbecco’s Modified Eagle’s Medium low glucose (DMEM); fetal bovine serum (FBS); penicillin/streptomycin, trypsin/EDTA; antimycotic) and Trypan Blue were all purchased from Gibco (Life Technologies do Brasil Ltda., São Paulo, SP, Brazil). 5-fluorouracil (Cas number 51–21-8), MTT (3-(4,5-Dimethylthiazol-2-yl)-2,5-diphenyltetrazolium bromide) (Cas number 298-93-1), propidium iodide (PI; Cat number 25535–16-4) and Dimethyl Sulfoxide (DMSO; Cas number 67685) were all purchased from Sigma-Aldrich (Merck KGaA, Darmstadt, Germany). Olaparib was purchased from Toronto Research Chemicals (Cat number 763113–22-0, Toronto Research Chemicals, North York, ON, Canada). PE Annexin V Apoptosis Detection Kit I was purchased from BD Bioscience (Cat number 559763, BD Bioscience, Franklin Lakes, NJ, USA). All other chemicals and solvents used were of analytical or pharmaceutical grade.

### Cell line and culture conditions

Two representative human colorectal cancer cell lines with different status of MMR proficiency, HCT116 (MSI, *MLH1*^*−/−*^ and *MSH3*^*−/−*^, *KRAS*^*mut*^, *TP53*^*wt*^) [34] and HT29 (MSS, *BRAF*^*mut*^ and *TP53*^*mut*^) [35] were originally obtained from ATTC® (Manassas, VA, USA). Cells were cultured in DMEM medium, supplemented with 10% FBS and 1% penicillin/streptomycin and antimycotic, incubated at 37 °C in humidified atmosphere with 5% CO_2_. Cultured cell concentrations were adjusted to allow for exponential growth. At 80% of confluence, cells were detached with 0.5% of trypsin/EDTA and used for the experiments.

### Experiment setup

Cells were seeded at different densities according to the experiment performed. After 24 h, cells were treated with 5-FU (5, 10, 20, 30 and 40 μM) or Olaparib (5 and 10 μM) or in the indicated combinations for 48 h or 72 h. Untreated cells were used as control. A 100 mM stock of 5-FU was prepared in absolute DMSO and stored at − 20 °C. Olaparib stock solution was prepared at 10 mM and stored at − 20 °C. Serial dilutions were prepared in culture medium.

### Cell viability

Cell viability was assessed by MTT assay. HCT116 and HT29 cells were adjusted to a density of 1 × 10^4^ cells and transferred to 96-well plate with a volume of 100 μL per well. After treatment, 100 μL of MTT solution (5 mg/mL) was added to each well and incubated at 37 °C for 3 h. Next, cell culture supernatant was removed and 100 μL of DMSO was added to each well to dissolve the formazan crystals. Absorbance readings of DMSO extracts were performed in SpectraMax® microplate reader (Molecular Devices, LLC, San Jose, CA, USA) at 540 nm.

The mode of interaction (synergy, antagonism or additivity) was determined by calculating the combination index (CI) [36] using CompuSyn software program (CompuSyn, Inc., Paramus, NJ). The degree of interaction between 5-FU and Olaparib was determined by the semiquantitative expression of ranges for antagonism (slight, if CI 1.1–1.2 to very strong, if CI > 10), synergism (slight, moderate, substantial, strong, and very strong), and additive interaction (CI 0.90–1.10) [37].

### Clonogenic survival

Cells were treated as previously described. After 48 h of exposure to treatments, cells were detached, counted and seeded in 6-well plates at a density of 6 × 10^2^ cells per well in complete medium. Therefore, cells were further incubated for 14 days to allow colony formation. Colonies were fixed with ice-cold methanol for 20 min and stained with crystal violet (0.5% w/v) solution for 10 min.

### Apoptosis determination

Assessment of cell death induction profile was performed as follows: HCT116 or HT29 cells were seeded in a density of 2 × 10^5^ cells in 6 well-plate (2 mL per well) and treated for 48 h or 72 h. Next, cells were detached and processed according to PE-Annexin V Apoptosis Detection Kit I manufacturer instructions (BD Biosciences). Cells were stained with PE-Annexin V and 7AAD at room temperature for 15 min in the dark. Finally, quantification of alive (Annexin^−^/7AAD^−^), early apoptotic (Annexin^+^/7AAD^−^) and late apoptotic/necrotic (Annexin^+/−^/7AAD^+^) cells was carried out on BD FACSCanto™ II flow cytometer (BD Bioscience, Franklin Lakes, NJ, USA) after 10,000 events acquisition.

### Cell cycle perturbation

Cells were seeded in 6 well-plates (2 × 10^5^ cells/well) and treated as previously described. After treatment, adherent and detached cells were fixed with chilled 80% ethanol at − 20 °C for at least 24 h. After removing ethanol, cells were blocked (0.1% TritonX-100in PBS), stained at 37 °C for 30 min in PBS containing 25 μg/mL PI and 50 μg/mL RNAse. Data was acquired in BD FACSCalibur™ flow cytometer (BD Biosciences, San Diego, CA, USA) after 20.000 events acquisition. The analysis was performed with CELLQuest software (BD Biosciences, San Diego, CA, USA).

### DNA damage induction

DNA damage induction was assessed by Comet Assay. Cells were seeded in 24 well-plates (1 × 10^4^ cells/well) and treated as previously described. At the end of treatment, HCT116 and HT29 cells were detached and 20 μL mixed with 100 μL 0.75% low melting point agarose at 37 °C and spread on the agarose-coated slides using a coverslip. Coverslips were removed after solidification at 4 °C for 3 min. The slides were immersed in cold lysis solution at 4 °C for 24 h. Next, slides were placed in an electrophoresis chamber filled with fresh alkaline buffer (300 mM NaOH, 1 mM EDTA, pH > 13) for 20 min at 4 °C. Electrophoresis was performed at 300 mA and 25 V for 20 min. The slides were washed 3 times with 10 mM Tris buffer (pH 7.5) for neutralization. Finally, the slides were stained with silver nitrate and analyzed at 200× magnification using an optical microscope. DNA damage was evaluated using visual classification from 0 (no damage, no tail) to 4 (maximum damage, long tails). One hundred cells were counted for each sample in duplicate slides. The DNA damage index was expressed in arbitrary units (A.U., 0–400).

### Adhesion measurements

Cells were treated for 48 h as previously described. Next, 3 × 10^2^ cells were then transferred to each well of a 24-well plate, and attached cells were counted on a light microscope following 2, 4, 6 and 24 h of seeding [38]. The adherence capacity (AC) was calculated as follows: AC = (number of attached/number of plated cells) × 100.

### Migration measurements

Cell motility was assessed in 8-μm pore polycarbonate membrane ThinCerts™ inserts (Greiner Bio-one Brazil, Americana, SP, Brazil). Cells were treated, detached, counted with trypan blue 0.4% and then 100 μL containing 2 × 10^5^ cells suspended in DMEM/FBS-free medium were added to the top of the insert, except for one, which contained DMEM/10% SFB as a negative control. In the insert bottom was added DMEM/20% FBS. Cells were allowed to migrate for 24 h. For quantification, non-migrated cells were removed with a swab, the membrane were fixed in methanol for 10 min and stained in Giemsa solution for 20 min. The membranes were washed with distilled water, removed with a scalpel and the bottom of the membrane was placed up in a slide. The membrane was covered by a coverslip. Migrated cells were counted in five different random fields.

### Statistical analyses

For data quantification, mean values and standard deviation (SD) or standard error of the mean (SEM) were calculated from at least three experimental replicates. Data were analyzed by Graphpad Prism, version 8.0 (Graphpad Instat – NIH, Bethesda, MD, USA) using one or two-way ANOVA (Analysis of Variance) according to the experimental design, followed by Tukey’s post-hoc. Two-sided *P*-values < 0.05 were considered statistically significant.

## Results

### Synergism of 5-FU and Olaparib is affected by MMR status in colorectal cancer

**Fig. 1:**
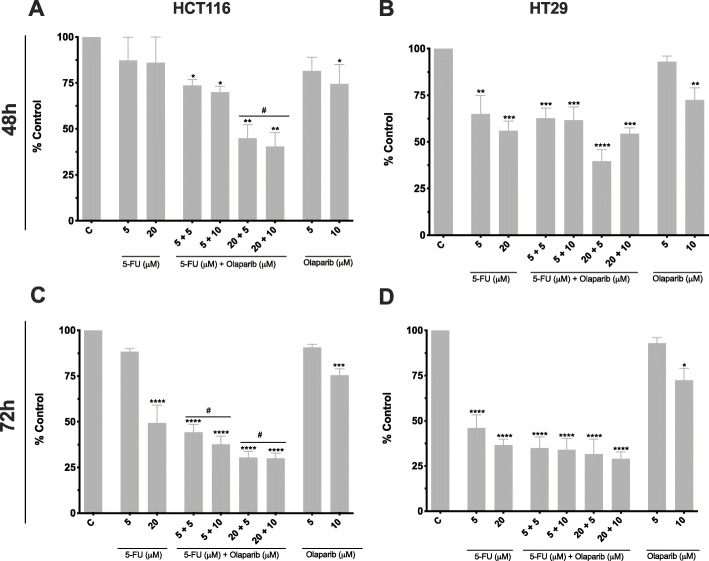
Viability assessment for treatment with 5-FU and Olaparib. **a, c** MTT assay of HCT116 cells treated with 5-FU and/or Olaparib for 48 h and 72 h, respectively (*n* = 3). **b, d** MTT assay of HT29 cells treated with 5-FU and/or Olaparib for 48 h and 72 h, respectively (*n* = 3). Untreated cells were used as negative control (c). One-way ANOVA and Tukey’s post-hoc tests. *p < 0.05, **p < 0.01, ***p < 0.001 and ****p < 0.0001 above error bars vs. control; ^#^*p* < 0.05 vs. 5-FU monotherapy in the same concentration

First, we examined the anti-proliferative properties of the two single drugs 5-FU and Olaparib and their combination in 1:1, 1:2, 4:1 and 2:1 mixtures by exposing MSI (HCT116) and MSS (Microsatellite stable) (HT29) CRC cell lines during 48 h or 72 h (Fig. 1). We observed a clear reduction of viability in HT29 cell line when treated with 5-FU alone, whereas in HCT116 a significant decrease was observed only for 72 h treatment at 20 μM 5-FU. A slight cytotoxic effect for treatment with 10 μM of Olaparib alone (mean reduction of 25%) was also observed. As expected, HT29 cells were more sensitive to 5-FU (IC_50_ = 36.7 μM, 48 h) than HCT116 (IC_50_ = 58.7 μM, 48 h), which is attributed to the requirement of proficient MMR to achieve 5-FU toxicity [7, 39]. In the combination, Olaparib increased the cytotoxic effect of 5-FU in HCT116 in a dose-dependent manner. While after 48 h of treatment, this effect was achieved only in combination to 20 μM of 5-FU (mean of 48% increment in cell death) (Fig. 1a), after prolonged exposure (72 h), it was also observed following exposure to Olaparib in combination to 5 μM of 5-FU (mean increment of 53% in cell death) (Fig. 1c). Conversely, sensitivity of HT29 to 5-FU was not significantly influenced by Olaparib addition (Fig. 1b and d).

**Fig. 2:**
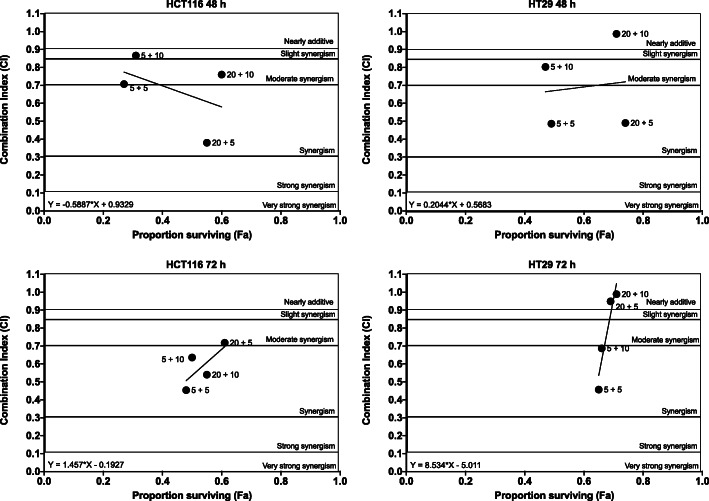
Interaction index plots of 5-FU and Olaparib in HCT116 and HT29 cells. Cells were treated with 5-FU and/or Olaparib for 48 h or 72 h, and then cell viability was measured using MTT assay. Interaction index plots were computed with the median-effect equation. The data of interaction plots are shown as the mean of all experiments (*n* = 3)

To determine whether the enhanced inhibitory effect observed in HCT116 cells following 5-FU and Olaparib combinations was additive or synergistic, we computed the combination index (CI) for the treatments using the aforementioned Chou [37]. As all interaction indexes of each 5-FU and Olaparib pair ranged from nearly additive to synergistic interaction (CI 0.3–0.7), we presented these ranges and the CI for each combination in Fig. 2**.** After 48 h treatment, the strongest synergistic inhibitory effect on HCT116 cell growth was reached with 20 μM 5-FU + 5 μM Olaparib, and in HT29 with 5 μM 5-FU + 5 μM Olaparib.

**Fig. 3:**
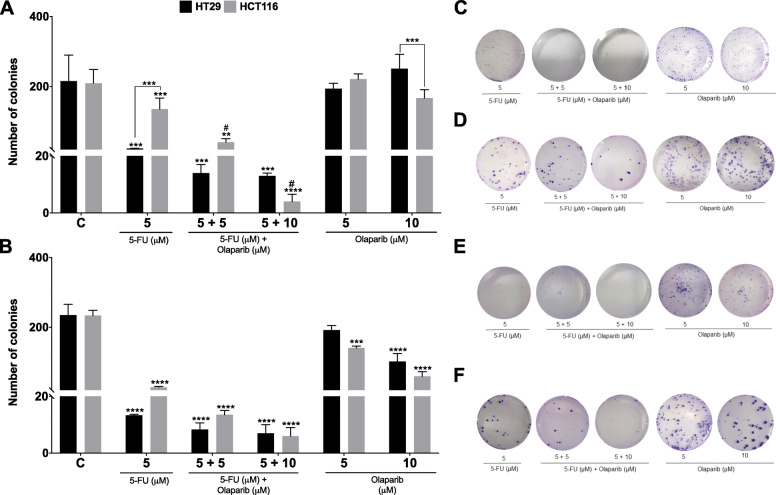
Clonogenic Survival Assay. Quantification of clonogenic survival assay for HT29 and HCT116 cell lines after 48 h (**a**) or 72 h (**b**) treatment with 5-FU and/or Olaparib (*n* = 3). Representative images of clonogenic survival assay for HT29 and HCT116 cell lines after 48 h (**c, d**) or 72 h (**e, f**) treatment with 5-FU and/or Olaparib, respectively. Untreated cells were used as negative control (C). One-way ANOVA and Tukey’s post-hoc tests. *p < 0.05, **p < 0.01, ***p < 0.001 and ****p < 0.0001 above error bars vs. control; ^#^*p* < 0.05 vs. 5-FU monotherapy in the same concentration

Some drugs may possibly cause injury which does not manifest until several cell generations when become lethal. Since the mechanisms by which synergistic effects of 5-FU and Olaparib may induce cell death include DNA damage, mitotic dysfunction and chromosome missegregation [40, 41], we investigated to what extent this combination affect clonogenic survival (Fig. 3). The ability of CRC cells treated for 48 h and 72 h to form colonies was evaluated after 14 days. In consonance to MTT assay results, HT29 cells were more sensitive than HCT116 to 5-FU alone with no further reduction of viability for the combination with Olaparib (Fig. 3). Addition of Olaparib to 5-FU for 48 h implicated in a reduction of 70% (5 μM 5-FU + 5 μM Olaparib) and 97% (5 μM 5-FU + 10 μM Olaparib) of the HCT116 ability to form colonies in relation to the 5-FU monotherapy (Fig. 3a). Also, 10 μM Olaparib monotherapy reduced the number of colony formation in both cell lines after 72 h (Fig. 3b).

**Fig. 4:**
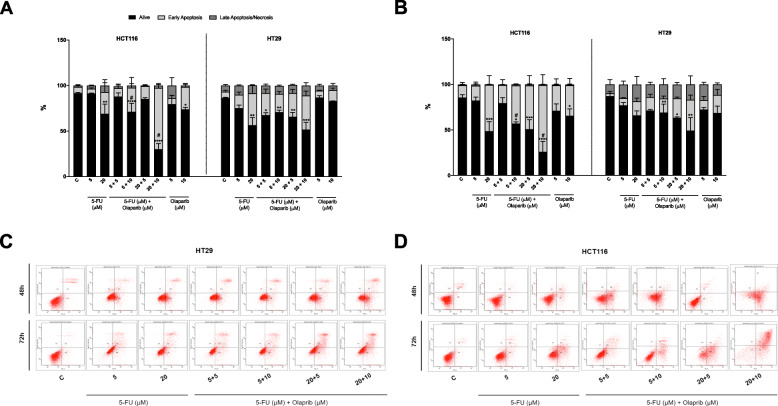
Apoptosis detection. Annexin-V and 7AAD staining was quantified by flow cytometry for HCT116 and HT29 treated with 5-FU and/or Olaparib for 48 h (**a**) or 72 h (**b**) (*n* = 3). Representative dotplots showing cell distribution between compartments in flow cytometry analysis using Annexin-V and 7-AAD staining for HT29 cells (**c**) or HCT116 cells (**d**) treated with 5-FU and/or Olaparib for 48 and 72 h. Untreated cells were used as negative control (c). One-way ANOVA and Tukey’s post-hoc tests. *p < 0.05, **p < 0.05, ***p < 0.001 and ****p < 0.0001 above error bars vs. control; ^#^*p* < 0.05 vs. 5-FU monotherapy in the same concentration

As a stronger synergistic anti-proliferative effect was observed following combination exposure to 5-FU and Olaparib in dMMR HCT116 cells, we aimed to determine whether the decreased cell survival was related to apoptosis. Combinations of 5 μM or 20 μM 5-FU + 10 μM Olaparib increased the population of HCT116 apoptotic cells after treatment of 48 h (increase of 21 and 38%, respectively) and 72 h (increase of 41 and 28%, respectively). However, none of the combinations enhanced the apoptotic population in HT29 cells in relation to the 5-FU alone at these time points (Fig. 4). Hence, our data suggest that, when Olaparib is combined with 5-FU, the apoptosis induction is enhanced in dMMR HCT116, but not in pMMR HT29 CRC cells. Interestingly, single administration of 10 μM Olaparib increased apoptosis in relation to the untreated control again only in HCT116 (Fig. 4) after 48 and 72 h.

### Olaparib treatment alone or in combination with 5-FU leads to generation of DNA strand breaks in MMR-proficient colon cancer cells

**Fig. 5:**
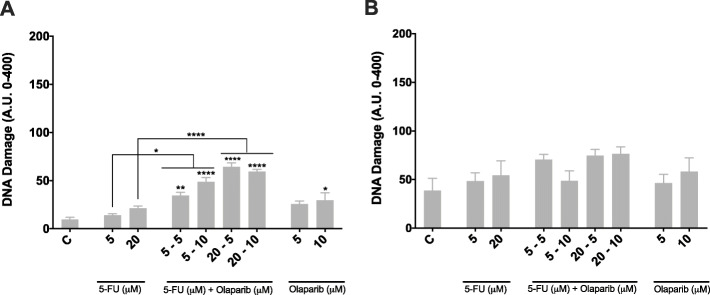
DNA damage index. DNA damage quantification by alkaline comet assay was performed immediately after 12 h of treatment with 5-FU and/or Olaparib in HT29 (**a**) and HCT116 (**b**) and cell lines (*n* = 3). Untreated cells were used as negative control. *p < 0.05, **p < 0.01, ***p < 0.001 and ****p < 0.0001 above error bars vs. control

To gain insights into the mechanisms underlying the apparent synergistic activity of 5-FU and Olaparib in the studied CRC cell lines, we examined the genome integrity using single cell gel electrophoresis (comet) assay. As can be seen on Fig. 5b no significant increase in the DNA strand breaks was observed in the MMR deficient HCT116 cell line. However, exposure of pMMR HT29 cells to 5-FU in combination with 5 or 10 μM of Olaparib induced DNA strand breaks after 12 h (Fig. 5a).

Unexpectedly, we identified higher basal DNA damage in HCT116 cells in comparison to HT29.

### Olaparib induces polyploidy and promotes G2/M arrest in the combined treatment with 5-FU in MMR-deficient colon cancer cells

**Fig. 6:**
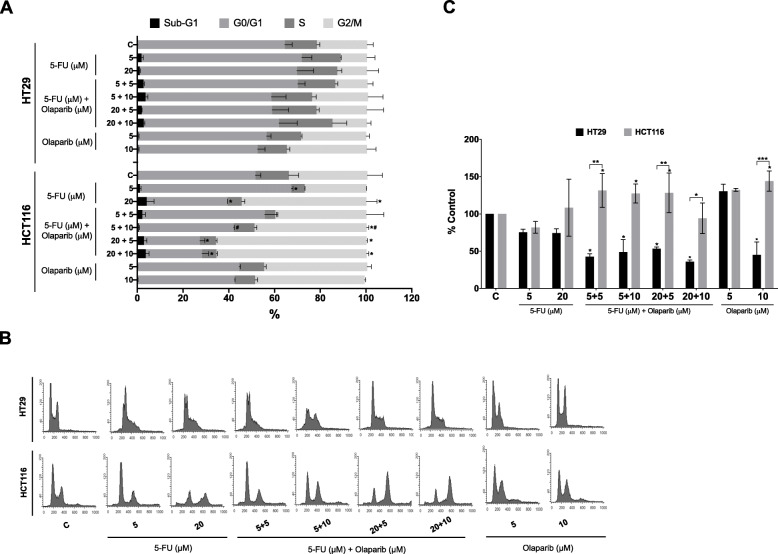
Cell cycle analysis. **a** Quantification of cell cycle profile analyzed by flow cytometry in HT29 and HCT116 cell lines after 48 h treatment with 5-FU and/or Olaparib (*n* = 3). *p < 0.05 above error bars vs. controls at the same cell cycle phase; #*p* < 0.05 vs. 5⎧M 5-FU in monotherapy. **b** Representative histograms of cell cycle profile in HT29 and HCT116 cell lines after 48 h treatment with 5-FU and/or Olaparib. **c** Polyploidy (>4n) population accumulation following 48 h treatment with 5-FU and/or Olaparib Untreated cells were used as negative control (c). *p < 0.05, **p < 0.01 and ***p < 0.001 above error bars vs. controls. Two-way ANOVA and Tukey’s post-hoc tests

Whereas the unrepaired DNA damage leads to senescence or apoptosis in drug-responsive cancer cells, loss of cell cycle checkpoints and increase of DNA damage tolerance allows subset of cancer cells perpetuate harmful mutations [42]. Therefore, we investigated whether cell cycle perturbations contributed to the cell death induction or growth inhibition by flow cytometry after 48 h of treatment (Fig. 6). It has been postulated that PARPis-induced cytotoxicity requires disruption of both replication and mitosis machinery. Replication blockage may be a consequence of entrapment and accumulation of inactive PARP1 as a result of PARP inhibition [43].

No significant cell cycle alterations are detected upon administration of 5-FU and Olaparib in HT29 CRC cells (Fig. 6a). Inversely, combined Olaparib and 5-FU treatment significantly increased the percentage of HCT116 cells in the G2/M phase in relation to the 5-FU alone (Fig. 6a). Strikingly, we detected a specific cell cycle interference following single Olaparib treatment, as a slight increase in the G2/M population of both cell lines and polyploidy induction only in MMR deficient HCT116 cells (Fig. 6c).

### Olaparib monotherapy impairs adhesion and migration capacities in MMR-proficient colon cancer cells

**Fig. 7:**
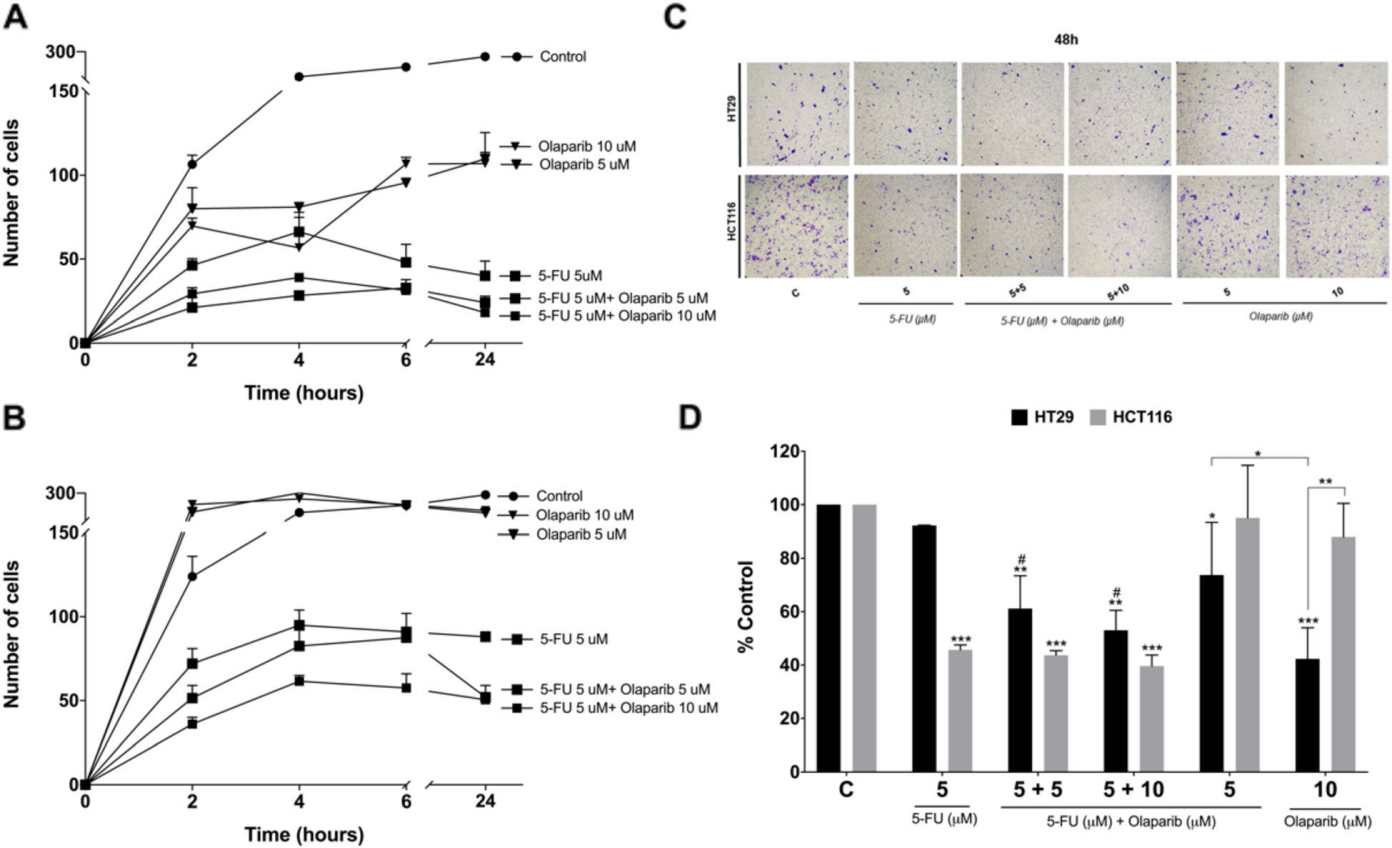
Adhesion and migration assays. Quantification of adherence capacity (number of attached cells) for (**a**) HT29 and (**b**) HCT116 cell lines after 48 h treatment with 5-FU and/or Olaparib (*n* = 3). *p < 0.05, **p < 0.01, ***p < 0.001 and ****p < 0.0001 next to symbols vs. untreated control at the same timepoint; ^#^*p* < 0.05 vs. 5-FU monotherapy in the same concentration at the same timepoint. **c** Representative images of migration capacity assay for HT29 and HCT116 cell lines after 48hs treatment with 5FU and/or Olaparib (*n* = 3). **d** Quantification of migrated cells for HT29 and HCT116 cell lines after 48hs treatment with 5FU and/or Olaparib. Untreated cells were used as negative control. *p < 0.05, **p < 0.01 and ***p < 0.001 above error bars vs. untreated control; ^#^*p* < 0.05 vs. 5-FU monotherapy in the same concentration. Two-way ANOVA and Tukey’s post hoc tests

Cell attachment is necessary for adherent cells to survive, proliferate and establish metastatic niches. For the best of our knowledge, this is the first study to explore the effects of 5-FU and Olaparib combination with regard to CRC cells adhesion and migration abilities. Thus, we treated both cell lines for 48 h and investigated adhesion capacity after 2, 4, 6, 12 and 24 h (Fig. 7a). Concerning HT29 cells, we observed two patterns, according to the reduction of cells attached after 24 h in comparison to controls: (1) intermediate (adhesion capacity was reduced to 50%): Olaparib treated cells; and (2) low (adhesion capacity was reduced to 30%): cells treated with 5 μM 5-FU alone or in combination with olaparib (Fig. 7a).

Unexpectedly, we observed that Olaparib monotherapy reduced adhesion capacity of HT29 cells, but no effect was exerted in HCT116 cells (Fig. 7b). In fact, this observation led us to investigate the metastatic potential of these cells by transwell assay (Fig. 7c and d). While 5 μM 5-FU did not affected HT29 cells migration ability, Olaparib addition to 5-FU resulted in a reduction of 30% in the number of migrated cells.

## Discussion

Conventional cancer regimens rely on chemotherapy, which generally exert its effects by inducing DNA damage or disrupting the mitotic machinery. While 5-FU has been the backbone of sporadic CRC treatment since 1960, advances in molecular comprehension, including the role of MMR deficiencies in response to therapy, brought to light a mechanism for 5-FU resistance. Alternatively, benefit may arise from combination therapy based on agents with non-overlapping mechanisms to reach synergistic or additive mechanism to overcome 5-FU resistance in the MMR deficient subset of colorectal tumors.

PARPis monotherapy has encountered its greatest efficacy in solid tumors with intrinsic HRR deficiencies. However, with the advances in the comprehension of PARPi sensitive-determining factors emerged the possibility of combinations with DNA damaging agents. Based on the fact that chemotherapy can increase DNA damage and PARPis can diminish the ability of PARP enzymes to repair DNA damage [44], it seems counterintuitive to explore these combinations. Thus, we hypothesized that causing DNA damage (via 5-FU) and impairing DNA damage response (via PARP inhibition) could be a potential strategy to recreate 5-FU sensitivity in colorectal tumors with MMR deficient background.

It has been postulated that apoptotic signaling cascade could be activated by the direct interaction of the damage-bound MMR heterodimers, hMutSα and hMutLα, and proteins of the ATR-Chk1 pathway. Consequently, it is thought that MMR acts as a DNA damage sensor, which has been referred as the “direct signaling model” [45, 46]. In this model, MMR role is beyond DNA repair, as it participates in signal transduction for cell death. Indeed, MMR protein-dependent pro-death signaling in response to various DNA-damaging agents have been well characterized, including 5-FU [11]. FU:G and FdUrd:G mismatches caused by 5-FU incorporation into DNA during replication are recognized by MSH2, inducing a futile cycle of repair [47]. Continuous attempts to daughter strand excision ultimately lead to disruption of DNA replication, which activates a MMR-dependent ATR-Chk1 signaling [48]. Contrariwise, in the absence of a functional MMR (particularly as a result of *hMLH1* loss*,* as in HCT116), caspase-3 cleavage does not occur and apoptosis cascade is not triggered [49].

Besides *hMLH1*, HCT116 cells also lack *hMSH3* and have low levels of ATM protein expression in comparison to other CRC cell lines [34, 35]. CRC harboring frameshift/nonsense mutation in *hMSH3* accounts for almost 50% of all MMR-deficient CRC [50], which brings clinical relevance to this molecular phenotype. Some *hMSH3*-deficient tumors behave like HRR-deficient tumors and present replication fork stress, since MSH3 is involved in DSBs and interstrand crosslinks (ICLs) repair [51]. Consequently, *hMSH3*-deficient tumors are potentially sensitive to PARPis [26]. However, few articles have investigated the effects of in vitro combinations of PARPis and 5-FU in colon cancer cell lines, with controversial results [31, 32].

When DNA damage occurs in the presence of a PARP inhibitor, such as Olaparib, PARP1 binds to damage sites and remains tightly bound or trapped onto the chromatin. That is whereby PARP inhibitors act as DNA poisons by trapping PARP on damaged DNA, resulting in cytotoxic PARP–DNA complexes. These complexes are much more cytotoxic than merely unrepaired SSBs, since PARylation is inhibited, and PARP1 remains bound to the lesion, which leads to stalled replication fork [24, 52]. The PARP1 cannot proceed with its role in fork stabilization and recruitment of the fork protection machinery [48, 53]. Consequently, replication fork collapses into DSBs, ultimately leading to cell death.

Our data for the combination treatment with 5-FU and Olaparib in the dMMR HCT116 cell line do not indicate increase in DNA strand breaks formation (Fig. 5b). So, the apoptosis induction in this cell line after PARP inhibition (Fig. 4b) should rely on other mechanism, different from replication fork collapse and DSBs formation. Instead, the observed accumulation of cells in G1 phase after 5 μM 5FU treatment, already described for the HCT116 cells [54], changes to G2 arrest after the combined treatment with 10 μM Olaparib (Fig. 6a). In this respect, it was shown that ATR inhibition potentiates cytotoxic effects of PARP inhibition by forced mitotic entry in cancer cells with deficient HRR [55]. The used single DNA fiber analysis in these circumstances showed that ATR inhibition does not increase replication fork degradation but accelerate mitotic entry, thus leading to chromatin bridges and lagging chromosomes formation. Moreover, in *BRCA*-mutant ovarian cancer cells, addition of ATRi or CHK1i released the cells from G2 phase arrest induced by PARPi causing premature mitotic entry resulting in increased chromosomal aberrations and apoptosis [56]. So, taken together our results in HCT116 cell line, having in mind its ATR-Chk1 signaling deficiency due to *hMLH1* loss, and the *hMSH3* mutation (i.e. HRR-deficient like phenotype), indicate that PARP inhibition leads to G2 arrest, premature mitosis entry and apoptosis induction in a combined treatment with 5-FU.

The 5-FU treatment sensitizes colorectal cancer cells towards DSBs and reduces HRR-mediated repair, which provide a biological rationale for its use as chemo- and radiosensitizer [13, 57]. While PARPis are synthetic lethal in HRR-deficient tumors, accumulating evidence suggests that PARylation is involved in both SSBs and DSBs repair [58]. However, whether MSI-tumors may also be susceptible to PARP inhibition is still a matter of discussion. Recently, a phase II trial reported that Olaparib monotherapy did not affect patient outcomes regardless of microsatellite status [59]. However, MSI-induced mutations in the DNA repair genes *MRE11* and *ATM* sensitizes gastrointestinal cancer cells to PARP inhibition [28, 60]. To date, chemical inhibition of PARP leads to replication fork collapse or accelerated fork progression that generates SSBs and DSBs [61, 62]. Since PARP1 promotes the repair of non-toxic single-strand DNA breaks, which are converted into potentially toxic DSBs during S-phase [63], the synergistic effect of the Olaparib and 5-FU cytotoxicity observed in HT29, could be attributed to increase in the DNA strand breaks induction.

While HT29 cells have chromosomal instability (CIN), HCT116 present MSI due to MMR deficiency. As aforementioned, HCT116 cells lack both *hMLH1* and *hMLH3*. It has been suggested that *hMLH3* plays a prominent role to elicit DSBs repair and DNA-damage response [26]. Actually, it was demonstrated that *hMSH3* silencing results in a 2-fold increase of DSBs [64]. In addition to the fact that MSI is associated with downregulation of MRE11 in colon cancer cells, which is essential for optimal signaling of ATM in response to DSBs [65], HCT116 cells also carry a mutation (c3380C > T) in one *ATM* allele, affecting ATM expression to half of normal [28]. This meets previous evidence in mantle cell lymphomas, gastric and colorectal tumors harboring mutations in *ATM* that result in loss or decrease of protein function and higher sensitivity to PARPis [28, 60, 66]. So, additional studies are needed in order to extend the observed synergistic effect of Olaparib and 5-FU to dMMR CRC cell lines with different genetic background.

While the increase in G2/M HT29 cells might be correlated with the DNA strand-breaks induction (Fig. 6a) and activation of DNA damage response, the mitotic arrest and polyploidy in HCT116 cells could be attributed to premature mitosis entry as discussed above. The cellular responses to mitotic delay are widely variable and appear to depend on an intriguing molecular competition between pathways leading to either apoptosis or slippage. Polyploid tumor cells are created by cytotoxic and targeted therapies, and elevated ploidy is likely to significantly increase during therapy [67]. However, since PARPis have been implicated in accumulation of polyploidy cells and in accordance to the hypothesis that polyploidy can result from mitotic defects, it may points out to a mechanism by which PARPis induce cell death [68]. By acting on replicating cells, Olaparib entraps PARP1 and obstructs replication fork progression, resulting in loss of sister chromatid cohesion in G2 cells [43]. Additionally, MLH1 (here functional only in HT29 cells) is known to be involved in DNA damage-induced checkpoint, favoring G2/M arrest [69].

Despite the low synergistic effect on cytotoxicity in MMR proficient HT29 cells, the combination of Olaparib and 5-FU increased DNA damage induction and decreased adhesion capacity of these cells. Cancer cells can undergo epithelial to mesenchymal transition (EMT), incorporating an invasive cell phenotype that can drive metastasis and enter a drug refractory state due to epigenetic reprograming. One of the mechanisms related to EMT facilitation is polyploidy, as giant polyploid tumor cells gain a mesenchymal phenotype [70]. In accordance to this concept, following Olaparib treatment in HT29 cells we observed the reduction of adhesion and migration capacities as at the same time the polyploid population accumulation was prevented. Contrariwise, HCT116 polyploid population was remarkably enriched after combination treatment with 5-FU and Olaparib, but without changes in the metastatic potential, which is already decreased by the 5-FU. Thus, if a possible MMR-status associated polyploidy induction in response to the treatment exits, it remains to be clarified in further investigations.

These observations are particularly relevant regarding a potential new role of Olaparib monotherapy in MMR proficient colorectal tumors. Despite we observed a reduction in cell viability and clonogenic survival following Olaparib monotherapy treatment irrespective to MSI status (Figs. 1 and 3), decrease of the adhesion and migration capacities was observed only in MMR proficient MSS cell line. Recent study, including 99 MSS cell lines did not show association between the sensitivity to Olaparib and specific consensus molecular subtype (CMS) or mutations in *KRAS* and *BRAF*, commonly accessed in CRC [71]. Moreover, two cell lines derived from different locations of the same patient, showed resistance (SW480, primary tumor) and sensitivity (SW620, lymph node metastasis) to Olaparib. However, most of the cell lines sensitive to Olaparib showed cross-sensitivity to oxaliplatin and non-functional HRR. Also, the data revealed that mutational signatures correlated to HR defects, BRCAness or HRR diagnostic assays do not completely discriminate PARPi susceptible CRC tumors but DNA repair functional tests. The cross-sensitivity between oxaliplatin and Olaparib was also confirmed in patient-derived organoids, and maintenance therapy with Olaparib was suggested for metastatic CRC patients with HRR deficient tumors that respond to first-line FOLFOX-chemotherapy [71]. Hence, Olaparib and 5-FU combination has stronger synergistic cytotoxic effect specifically to MMR deficient MSI CRC, while Olaparib monotherapy may emerge as a maintenance therapy for unresectable and metastatic MMR proficient MSS CRC.

## Conclusions

Here, we provide a rational for the inclusion of the PARP inhibitor Olaparib in two new therapeutic approaches for CRC: (1) in combination with 5-FU for dMMR/MSI CRC and (2) as monotherapy for pMMR/MSS CRC. Our results showed the 5-FU cytotoxicity enhancement by Olaparib in dMMR HCT116 colon cancer cells. The observed synergistic effect of the combined treatment in these cells could be attributed to impaired adhesion ability and apoptosis induction. This is particularly relevant since MMR-deficient cells are more resistant to 5-FU, and Olaparib addition can result in 5-FU dose reduction. Although, further studies are needed to confirm the observed effect in dMMR CRC cell lines with different genetic background. For the first time, we observed DNA damage induction and impairment of adhesion and migration capacities following Olaparib in monotherapy treatment only in MMR proficient cells. Therefore, the combined Olaparib and 5-FU treatment could benefit patients with non-metastatic dMMR/MSI colorectal tumors, while Olaparib monotherapy may have a role as a maintenance therapy for advanced MMR proficient/MSS CRC.

## Data Availability

The datasets used and/or analysed during the current study are available from the corresponding author on reasonable request.

## Abbreviations

5-FU: 5-fluorouracil (5-FU)
BER: Base Excision Repair
CI: Combination index
CIN: Chromosomal instability
CRC: Colorectal cancer
dMMR: MMR deficient pMMR MMR proficient
DSBs: Double-stranded DNA breaks
EMT: Epithelial to mesenchymal transition
h: Hours
HRR: Homologous Recombination Repair
ICLs: Interstrand crosslinks
MMR: Mismatch Repair
MSI: Microsatellite Instability
MSS: Microsatellite stable
PARP1: Poly-(ADP-ribose) polymerase 1
PARPis: PARP inhibitors
pMMR: Proficient MMR
SSBs: Single-strand DNA breaks
TS: Thymidylate synthase
UDG: Uracil–DNA glycosylase
