# Correction: Olaparib-mediated enhancement of 5-fluorouracil cytotoxicity in mismatch repair deficient colorectal cancer cells

**DOI:** 10.1186/s12885-026-16371-x

**Published:** 2026-07-01

**Authors:** Helena de Castro e Gloria, Laura Jesuíno Nogueira, Patrícia Bencke Grudzinski, Paola Victória da Costa Ghignatti, Temenouga Nikolova Guecheva, Natalia Motta Leguisamo, Jenifer Saffi

**Affiliations:** 1 Laboratory of Genetic Toxicology, Federal University of Health Sciences of Porto Alegre (UFCSPA), Sarmento Leite st 245, Porto Alegre, RS Brazil; 2 Cardiology Institute of Rio Grande do Sul/ University Foundation of Cardiology (ICFUC), Porto Alegre, RS Brazil

**Correction: BMC Cancer 21**,** 448 (2021)**

**https://doi.org/10.1186/s12885-021-08188-7**

Following publication of the original article [1], the author reported that an image duplication was identified in Fig. 7C. Specifically, the image corresponding to the HCT116 5-FU 5 µM + Olaparib 5 µM condition was inadvertently duplicated and incorrectly used in place of the HCT116 5-FU 5 µM condition.

As the quantitative analysis was performed directly on the original sample slides, independently of the representative images selected for illustration, the replacement of the image does not affect the reported results and conclusions.

The incorrect image is.


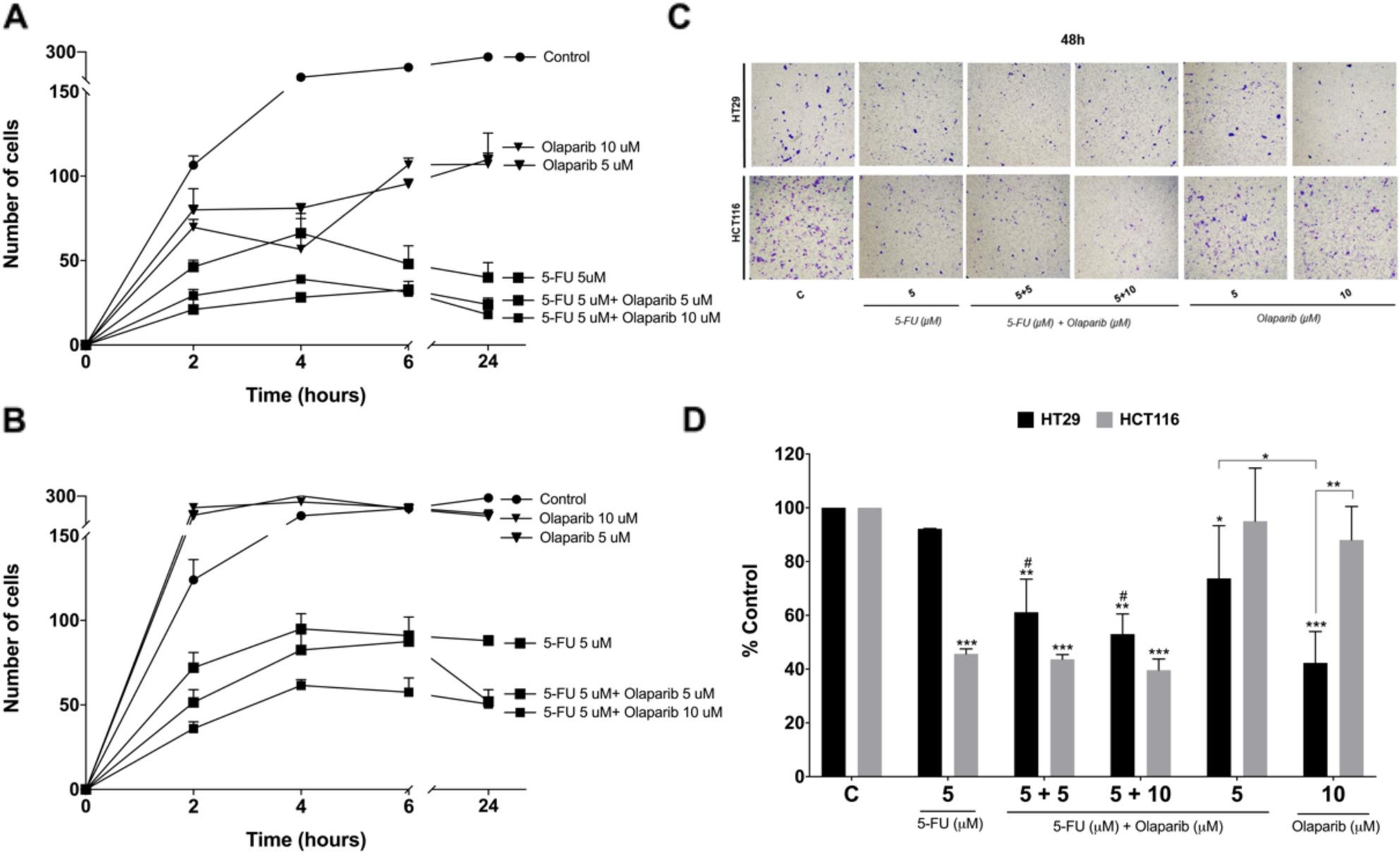


The correct image is.


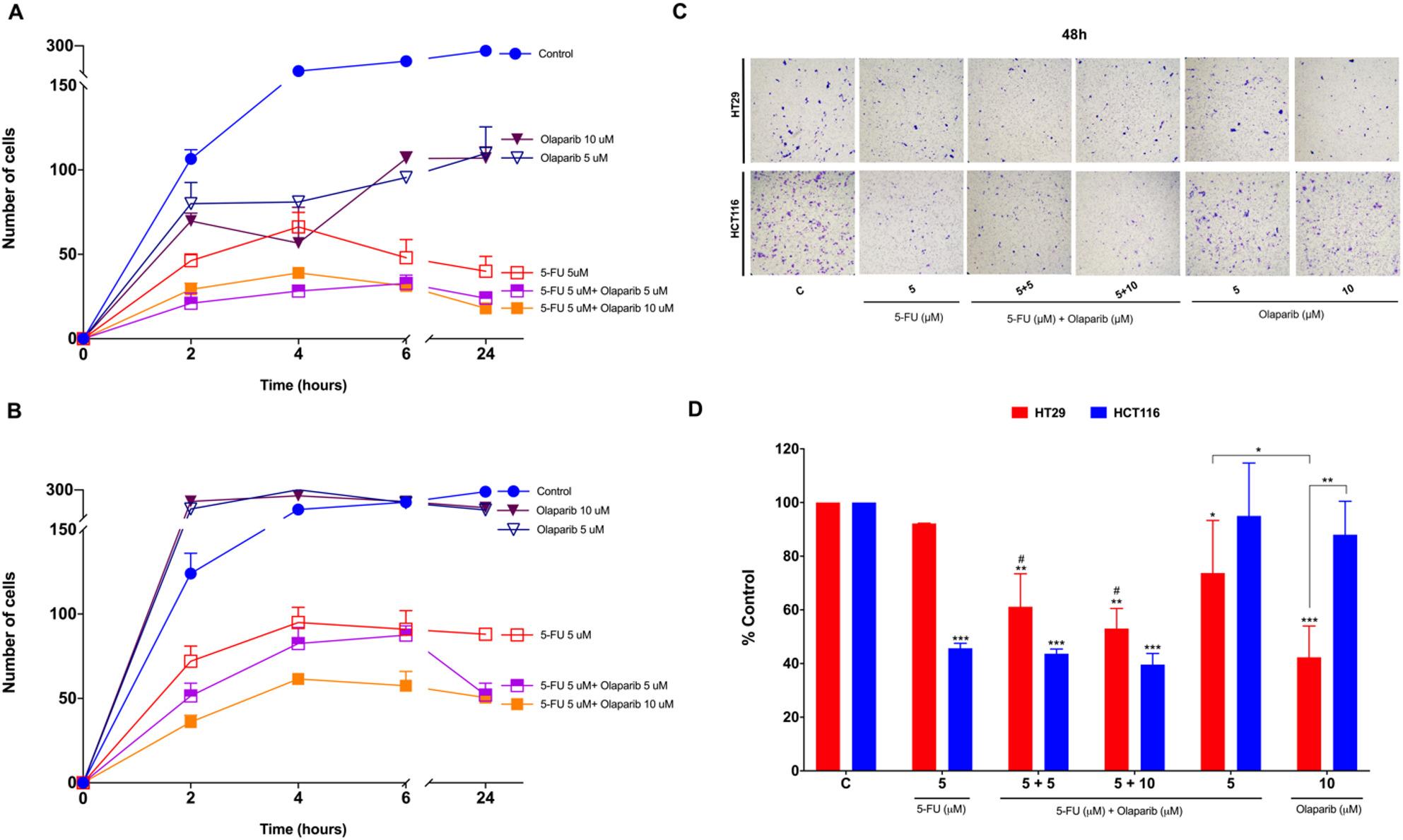


The original article has been updated.
